# Supplementary material for: Modeling glioblastoma heterogeneity as a dynamic network of cell states
Source: Mol Syst Biol. 2021 Sep 16;17(9):e10105. doi: 10.15252/msb.202010105 (PMC8444284; doi:10.15252/msb.202010105)
Supplement: Supplementary file 6 — Source Data for Figure 5 [file MSB-17-e10105-s004.zip › Figure5A_sourcedata/GSEA_3017/nosigdb_stateA.GseaPreranked.1622795026485/RICHARDS_NOWAKOWSKI_SCIENCE_2017_IPC_DIV1_UPREG.html]

Details for gene set RICHARDS\_NOWAKOWSKI\_SCIENCE\_2017\_IPC\_DIV1\_UPREG[GSEA]

|  || Dataset | state53017 |
| Phenotype | NoPhenotypeAvailable |
| Upregulated in class | na\_pos |
| GeneSet | RICHARDS\_NOWAKOWSKI\_SCIENCE\_2017\_IPC\_DIV1\_UPREG |
| Enrichment Score (ES) | 0.38193145 |
| Normalized Enrichment Score (NES) | 3.3057282 |
| Nominal p-value | 0.0 |
| FDR q-value | 0.0 |
| FWER p-Value | 0.0 |
Table: GSEA Results Summary

  

Fig 1: Enrichment plot: RICHARDS\_NOWAKOWSKI\_SCIENCE\_2017\_IPC\_DIV1\_UPREG      
 Profile of the Running ES Score & Positions of GeneSet Members on the Rank Ordered List

  

| PROBE | GENE SYMBOL | GENE\_TITLE | RANK IN GENE LIST | RANK METRIC SCORE | RUNNING ES | CORE ENRICHMENT || 1 | CDK1 |  |  | 3 | 1.006 | 0.0099 | Yes |
| 2 | MKI67 |  |  | 4 | 0.926 | 0.0224 | Yes |
| 3 | KIF11 |  |  | 6 | 0.870 | 0.0329 | Yes |
| 4 | UBE2C |  |  | 7 | 0.793 | 0.0436 | Yes |
| 5 | MCM10 |  |  | 9 | 0.752 | 0.0525 | Yes |
| 6 | KIF20B |  |  | 10 | 0.749 | 0.0626 | Yes |
| 7 | CEP55 |  |  | 11 | 0.747 | 0.0727 | Yes |
| 8 | BUB3 |  |  | 12 | 0.740 | 0.0828 | Yes |
| 9 | KIF5B |  |  | 16 | 0.687 | 0.0883 | Yes |
| 10 | KPNA2 |  |  | 20 | 0.667 | 0.0936 | Yes |
| 11 | HIST1H4C |  |  | 21 | 0.659 | 0.1025 | Yes |
| 12 | ASPM |  |  | 27 | 0.639 | 0.1049 | Yes |
| 13 | DANCR |  |  | 29 | 0.632 | 0.1122 | Yes |
| 14 | PIF1 |  |  | 30 | 0.627 | 0.1207 | Yes |
| 15 | DNAJC9 |  |  | 34 | 0.615 | 0.1253 | Yes |
| 16 | PLCB1 |  |  | 36 | 0.604 | 0.1322 | Yes |
| 17 | CCNB1 |  |  | 37 | 0.599 | 0.1403 | Yes |
| 18 | CKS2 |  |  | 40 | 0.587 | 0.1458 | Yes |
| 19 | HMMR |  |  | 43 | 0.559 | 0.1508 | Yes |
| 20 | NCAPG |  |  | 44 | 0.556 | 0.1584 | Yes |
| 21 | HJURP |  |  | 47 | 0.539 | 0.1632 | Yes |
| 22 | CKS1B |  |  | 50 | 0.530 | 0.1678 | Yes |
| 23 | DBF4 |  |  | 55 | 0.514 | 0.1698 | Yes |
| 24 | SMC3 |  |  | 58 | 0.504 | 0.1742 | Yes |
| 25 | KIF14 |  |  | 59 | 0.503 | 0.1809 | Yes |
| 26 | MASTL |  |  | 60 | 0.499 | 0.1877 | Yes |
| 27 | CENPE |  |  | 62 | 0.496 | 0.1931 | Yes |
| 28 | MIS18BP1 |  |  | 64 | 0.495 | 0.1986 | Yes |
| 29 | CCNA2 |  |  | 65 | 0.495 | 0.2053 | Yes |
| 30 | TPX2 |  |  | 66 | 0.488 | 0.2119 | Yes |
| 31 | DLGAP5 |  |  | 76 | 0.475 | 0.2071 | Yes |
| 32 | HIST1H1B |  |  | 77 | 0.475 | 0.2135 | Yes |
| 33 | AURKA |  |  | 78 | 0.474 | 0.2199 | Yes |
| 34 | CDC20 |  |  | 80 | 0.472 | 0.2251 | Yes |
| 35 | VRK1 |  |  | 82 | 0.470 | 0.2302 | Yes |
| 36 | UBE2T |  |  | 83 | 0.470 | 0.2365 | Yes |
| 37 | PLK1 |  |  | 89 | 0.464 | 0.2366 | Yes |
| 38 | TUBB4B |  |  | 93 | 0.462 | 0.2391 | Yes |
| 39 | CENPF |  |  | 94 | 0.461 | 0.2454 | Yes |
| 40 | TOP2A |  |  | 95 | 0.457 | 0.2515 | Yes |
| 41 | HIST1H1D |  |  | 98 | 0.456 | 0.2552 | Yes |
| 42 | ZWINT |  |  | 106 | 0.447 | 0.2526 | Yes |
| 43 | TGIF1 |  |  | 107 | 0.447 | 0.2586 | Yes |
| 44 | AURKB |  |  | 108 | 0.446 | 0.2646 | Yes |
| 45 | ZEB1 |  |  | 109 | 0.446 | 0.2706 | Yes |
| 46 | SEPHS1 |  |  | 114 | 0.437 | 0.2716 | Yes |
| 47 | CDCA8 |  |  | 115 | 0.437 | 0.2775 | Yes |
| 48 | KIF23 |  |  | 116 | 0.436 | 0.2834 | Yes |
| 49 | DEPDC1 |  |  | 119 | 0.432 | 0.2867 | Yes |
| 50 | MELK |  |  | 123 | 0.427 | 0.2888 | Yes |
| 51 | BUB1B |  |  | 129 | 0.421 | 0.2883 | Yes |
| 52 | HELLS |  |  | 135 | 0.417 | 0.2877 | Yes |
| 53 | RTKN2 |  |  | 141 | 0.413 | 0.2871 | Yes |
| 54 | GTSE1 |  |  | 146 | 0.411 | 0.2877 | Yes |
| 55 | CCNB2 |  |  | 147 | 0.411 | 0.2932 | Yes |
| 56 | ANLN |  |  | 152 | 0.407 | 0.2938 | Yes |
| 57 | MAD2L1 |  |  | 153 | 0.406 | 0.2992 | Yes |
| 58 | LINC01224 |  |  | 154 | 0.406 | 0.3047 | Yes |
| 59 | KIFC1 |  |  | 160 | 0.402 | 0.3039 | Yes |
| 60 | ARHGAP11A |  |  | 162 | 0.399 | 0.3081 | Yes |
| 61 | GNG4 |  |  | 164 | 0.398 | 0.3122 | Yes |
| 62 | KIF2C |  |  | 165 | 0.397 | 0.3176 | Yes |
| 63 | MYC |  |  | 169 | 0.395 | 0.3192 | Yes |
| 64 | CCAR1 |  |  | 170 | 0.395 | 0.3246 | Yes |
| 65 | NDC80 |  |  | 173 | 0.393 | 0.3274 | Yes |
| 66 | FAM83D |  |  | 180 | 0.389 | 0.3252 | Yes |
| 67 | HNRNPH3 |  |  | 185 | 0.387 | 0.3255 | Yes |
| 68 | ARHGAP19 |  |  | 187 | 0.385 | 0.3294 | Yes |
| 69 | BUB1 |  |  | 188 | 0.384 | 0.3346 | Yes |
| 70 | DCLRE1C |  |  | 190 | 0.381 | 0.3386 | Yes |
| 71 | KIF22 |  |  | 197 | 0.377 | 0.3362 | Yes |
| 72 | CDCA3 |  |  | 203 | 0.372 | 0.3350 | Yes |
| 73 | SUV39H2 |  |  | 205 | 0.371 | 0.3388 | Yes |
| 74 | PTMA |  |  | 206 | 0.371 | 0.3438 | Yes |
| 75 | LRRN1 |  |  | 210 | 0.370 | 0.3451 | Yes |
| 76 | ATCAY |  |  | 215 | 0.368 | 0.3451 | Yes |
| 77 | ACTR1A |  |  | 216 | 0.367 | 0.3501 | Yes |
| 78 | SMC4 |  |  | 217 | 0.367 | 0.3550 | Yes |
| 79 | SPC25 |  |  | 226 | 0.363 | 0.3500 | Yes |
| 80 | INCENP |  |  | 227 | 0.362 | 0.3549 | Yes |
| 81 | NCAPH |  |  | 232 | 0.359 | 0.3548 | Yes |
| 82 | CENPA |  |  | 243 | 0.352 | 0.3471 | Yes |
| 83 | H2AFZ |  |  | 245 | 0.351 | 0.3507 | Yes |
| 84 | TTK |  |  | 262 | 0.340 | 0.3354 | Yes |
| 85 | KNSTRN |  |  | 263 | 0.340 | 0.3400 | Yes |
| 86 | TUBB |  |  | 264 | 0.340 | 0.3446 | Yes |
| 87 | SLC25A5 |  |  | 266 | 0.340 | 0.3479 | Yes |
| 88 | CENPJ |  |  | 270 | 0.339 | 0.3488 | Yes |
| 89 | CLSPN |  |  | 274 | 0.336 | 0.3496 | Yes |
| 90 | DEPDC1B |  |  | 275 | 0.335 | 0.3541 | Yes |
| 91 | ESCO2 |  |  | 282 | 0.333 | 0.3512 | Yes |
| 92 | ORC6 |  |  | 283 | 0.333 | 0.3557 | Yes |
| 93 | C21orf58 |  |  | 294 | 0.328 | 0.3477 | Yes |
| 94 | C2orf69 |  |  | 297 | 0.326 | 0.3496 | Yes |
| 95 | G2E3 |  |  | 302 | 0.325 | 0.3491 | Yes |
| 96 | MND1 |  |  | 304 | 0.324 | 0.3522 | Yes |
| 97 | RAD18 |  |  | 307 | 0.324 | 0.3541 | Yes |
| 98 | BRCA2 |  |  | 308 | 0.323 | 0.3585 | Yes |
| 99 | CENPN |  |  | 310 | 0.323 | 0.3616 | Yes |
| 100 | FANCI |  |  | 319 | 0.319 | 0.3560 | Yes |
| 101 | DNA2 |  |  | 321 | 0.318 | 0.3590 | Yes |
| 102 | TUBA1C |  |  | 322 | 0.318 | 0.3633 | Yes |
| 103 | STAM |  |  | 325 | 0.317 | 0.3651 | Yes |
| 104 | RFC3 |  |  | 337 | 0.314 | 0.3557 | Yes |
| 105 | CDCA5 |  |  | 341 | 0.312 | 0.3562 | Yes |
| 106 | CLIC1 |  |  | 342 | 0.311 | 0.3604 | Yes |
| 107 | CHD7 |  |  | 343 | 0.311 | 0.3646 | Yes |
| 108 | PRC1 |  |  | 350 | 0.310 | 0.3614 | Yes |
| 109 | ANP32B |  |  | 357 | 0.307 | 0.3581 | Yes |
| 110 | MRPS18A |  |  | 359 | 0.306 | 0.3610 | Yes |
| 111 | ECT2 |  |  | 365 | 0.305 | 0.3589 | Yes |
| 112 | CCNF |  |  | 366 | 0.305 | 0.3630 | Yes |
| 113 | TTF2 |  |  | 367 | 0.305 | 0.3671 | Yes |
| 114 | CDCA2 |  |  | 369 | 0.304 | 0.3700 | Yes |
| 115 | MYEF2 |  |  | 371 | 0.303 | 0.3728 | Yes |
| 116 | BRCA1 |  |  | 373 | 0.302 | 0.3757 | Yes |
| 117 | CKAP2 |  |  | 379 | 0.301 | 0.3735 | Yes |
| 118 | RRM2 |  |  | 383 | 0.300 | 0.3739 | Yes |
| 119 | XRCC2 |  |  | 384 | 0.300 | 0.3779 | Yes |
| 120 | CENPW |  |  | 386 | 0.299 | 0.3807 | Yes |
| 121 | PHF19 |  |  | 393 | 0.297 | 0.3773 | Yes |
| 122 | RACGAP1 |  |  | 399 | 0.293 | 0.3750 | Yes |
| 123 | PA2G4 |  |  | 402 | 0.293 | 0.3765 | Yes |
| 124 | HMGB1 |  |  | 403 | 0.292 | 0.3804 | Yes |
| 125 | CCT5 |  |  | 409 | 0.290 | 0.3782 | Yes |
| 126 | NFIA |  |  | 413 | 0.289 | 0.3784 | Yes |
| 127 | DCP2 |  |  | 416 | 0.289 | 0.3798 | Yes |
| 128 | HMGB2 |  |  | 424 | 0.288 | 0.3750 | Yes |
| 129 | NUSAP1 |  |  | 430 | 0.286 | 0.3726 | Yes |
| 130 | NUF2 |  |  | 434 | 0.285 | 0.3728 | Yes |
| 131 | MIS18A |  |  | 439 | 0.284 | 0.3716 | Yes |
| 132 | NCAPD2 |  |  | 440 | 0.283 | 0.3755 | Yes |
| 133 | LMNB1 |  |  | 442 | 0.282 | 0.3780 | Yes |
| 134 | CDC25C |  |  | 443 | 0.282 | 0.3819 | Yes |
| 135 | KIF20A |  |  | 447 | 0.281 | 0.3819 | Yes |
| 136 | ANP32E |  |  | 453 | 0.280 | 0.3795 | No |
| 137 | SMS |  |  | 463 | 0.278 | 0.3721 | No |
| 138 | ATAD2 |  |  | 470 | 0.275 | 0.3684 | No |
| 139 | EZH2 |  |  | 471 | 0.275 | 0.3721 | No |
| 140 | SKA1 |  |  | 475 | 0.274 | 0.3721 | No |
| 141 | SHCBP1 |  |  | 477 | 0.274 | 0.3745 | No |
| 142 | EFS |  |  | 487 | 0.272 | 0.3671 | No |
| 143 | BLM |  |  | 490 | 0.272 | 0.3682 | No |
| 144 | KIF4A |  |  | 504 | 0.269 | 0.3558 | No |
| 145 | NUP93 |  |  | 506 | 0.269 | 0.3582 | No |
| 146 | HAUS8 |  |  | 518 | 0.267 | 0.3481 | No |
| 147 | RCC1 |  |  | 528 | 0.265 | 0.3405 | No |
| 148 | SKA3 |  |  | 531 | 0.264 | 0.3416 | No |
| 149 | KIF15 |  |  | 538 | 0.263 | 0.3377 | No |
| 150 | CDKN3 |  |  | 539 | 0.263 | 0.3413 | No |
| 151 | STIL |  |  | 545 | 0.261 | 0.3386 | No |
| 152 | CENPO |  |  | 549 | 0.260 | 0.3384 | No |
| 153 | CCDC77 |  |  | 553 | 0.259 | 0.3382 | No |
| 154 | FBXO5 |  |  | 557 | 0.258 | 0.3379 | No |
| 155 | SPDL1 |  |  | 560 | 0.258 | 0.3389 | No |
| 156 | DDX39A |  |  | 562 | 0.258 | 0.3412 | No |
| 157 | ZMIZ1 |  |  | 573 | 0.255 | 0.3322 | No |
| 158 | TMPO |  |  | 584 | 0.253 | 0.3232 | No |
| 159 | ZNF714 |  |  | 594 | 0.251 | 0.3154 | No |
| 160 | FAM111A |  |  | 618 | -0.259 | 0.2904 | No |
| 161 | TACC1 |  |  | 623 | -0.260 | 0.2890 | No |
| 162 | MAGI1 |  |  | 642 | -0.266 | 0.2702 | No |
| 163 | EML1 |  |  | 649 | -0.269 | 0.2664 | No |
| 164 | SORT1 |  |  | 664 | -0.279 | 0.2528 | No |
| 165 | DCTN3 |  |  | 665 | -0.280 | 0.2566 | No |
| 166 | CKB |  |  | 676 | -0.287 | 0.2481 | No |
| 167 | CNR1 |  |  | 695 | -0.298 | 0.2298 | No |
| 168 | CUX1 |  |  | 775 | -0.364 | 0.1367 | No |
| 169 | NKAIN3 |  |  | 808 | -0.389 | 0.1022 | No |
| 170 | POU3F2 |  |  | 843 | -0.437 | 0.0659 | No |
| 171 | MDM2 |  |  | 847 | -0.442 | 0.0682 | No |
| 172 | TMSB4X |  |  | 850 | -0.443 | 0.0717 | No |
| 173 | TFDP2 |  |  | 858 | -0.448 | 0.0691 | No |
| 174 | EZR |  |  | 865 | -0.464 | 0.0679 | No |
| 175 | DAAM1 |  |  | 885 | -0.516 | 0.0513 | No |
| 176 | GPC6 |  |  | 896 | -0.540 | 0.0462 | No |
| 177 | ENC1 |  |  | 922 | -0.621 | 0.0235 | No |
| 178 | FRMD4B |  |  | 925 | -0.626 | 0.0295 | No |
| 179 | EFNB2 |  |  | 936 | -0.682 | 0.0263 | No |
| 180 | NFIX |  |  | 940 | -0.692 | 0.0319 | No |
| 181 | CSRP2 |  |  | 960 | -0.877 | 0.0202 | No |
| 182 | TAGLN2 |  |  | 963 | -0.891 | 0.0298 | No |
Table: GSEA details [plain text format]

  

Fig 2: RICHARDS\_NOWAKOWSKI\_SCIENCE\_2017\_IPC\_DIV1\_UPREG: Random ES distribution      
 Gene set null distribution of ES for **RICHARDS\_NOWAKOWSKI\_SCIENCE\_2017\_IPC\_DIV1\_UPREG**
